# Skewed pulmonary innate immune cell composition underlies the delayed influenza clearance in aged mice

**DOI:** 10.3389/fmicb.2025.1734163

**Published:** 2025-12-10

**Authors:** Xiaoyang Cheng, Fang Zhao, Jing Wu, Yanmin Wan, Zhaoqin Zhu, Jialin Jin

**Affiliations:** 1 Department of Infectious Diseases, Shanghai Key Laboratory of Infectious Diseases and Biosafety Emergency Response, National Medical Center for Infectious Diseases, Huashan Hospital, Shanghai Medical College, Fudan University, Shanghai, China; 2 Department of Laboratory Medicine, Shanghai Public Health Clinical Center, Shanghai, China; 3 Department of Radiology, Shanghai Xuhui Central Hospital, Shanghai, China; 4 Department of Radiology, Shanghai Public Health Clinical Center, Shanghai, China; 5 Shanghai Sci-Tech Inno Center for Infection and Immunity, Shanghai, China

**Keywords:** aging, influenza, sublethal infection, innate immune cell, inflammatory cytokine

## Abstract

**Introduction:**

Aging increases vulnerability of the elderly to influenza, but the mechanisms have not been fully understood. Although lethally infected aged mice are frequently used as models of influenza infection in the elderly, they are more suitable for studying mortality rather than the increased disease severity frequently observed in non-lethal infections. Therefore, understanding age-related differences under sublethal infection conditions is crucial.

**Methods:**

Adult (8–12 months) and aged (22–24 months) mice were infected with a sublethal dose of H1N1 (PR8). Body weight loss, lung viral titers, pulmonary innate immune cell composition, and transcriptional levels of key inflammatory cytokines were assessed.

**Results:**

Despite similar body weight loss, the aged mice showed significantly higher lung viral titers at day 8. Notably, key innate immune populations, including alveolar macrophages, neutrophils, and eosinophils, showed distinct age-related patterns. In the adult mice, alveolar macrophages negatively correlated with weight loss, whereas no protective immune factor was identified in the aged mice. Moreover, our data showed that persistent viral replication led to distinct innate immune cell composition in the adult and aged mice despite comparable transcription levels of inflammatory cytokines. The numbers and frequencies of both the neutrophils and eosinophils were significantly higher in the virus-persistent adult mice than those in the virus-persistent aged mice.

**Discussion:**

Our findings reveal skewed acute immune responses to influenza in aged mice, which may partially account for their mild weight loss despite delayed viral clearance and highlight age-related impairments in early antiviral immunity.

## 1 Introduction

Influenza remains a major global cause of seasonal morbidity and mortality, disproportionately affecting elderly populations (Centers for Disease Control and Prevention, 2024). Epidemiological studies have shown that advanced age is associated with increased susceptibility to influenza, more severe clinical manifestations, delayed recovery, and elevated mortality risk (Nguyen and Noymer, 2013; GBD 2017 Influenza Collaborators, 2019; Cipelli et al., 2024). It was estimated that 67% of influenza-related respiratory deaths were among the elder population aged 65 years and older (Paget et al., 2019). Meanwhile, the efficacy of influenza vaccine is markedly reduced in the elderly (Murasko et al., 2002; Cerqueira-Silva et al., 2022). A recent meta-analysis of real-world data suggested that influenza vaccine effectiveness decreased significantly among people aged 65 years and older especially when compared to children (Guo et al., 2024). Immunosenescence,—an age associated decline of immune function (Haq and McElhaney, 2014; Allen et al., 2020; Liu et al., 2023), is thought to be a major player that contributes to the impaired infection control and decreased vaccine efficacy (Lee et al., 2022; Doherty et al., 2025). Despite continuous efforts, the underlying mechanism has not been fully understood (Yang et al., 2025), partly because the impact of aging on infection and vaccine effectiveness is far more complex than mere immunosenescence. Two recent studies tried to deconvolute the aging associated changes in influenza disease severity and vaccine effectiveness (Riese et al., 2022; Kasmani et al., 2023), which uncovered complicated cellular and molecular alterations in aged mice or individuals upon influenza infection or vaccination using high throughput sequencing and immunological methods.

In addition to the sophisticated nature of aging, the setup of influenza-infected aging animal model is also an important limitation for researches in this field. Mouse is one of the most frequently used models in aging research (Holtze et al., 2021). Most previous studies employed lethally infected aged mouse models to investigate age-related changes during influenza infection, which provided important insights into disease severity, delayed viral clearance, impaired immune responses, and enhanced lung pathology associated with immunosenescence (Wong et al., 2017; Chen et al., 2022; Jiang et al., 2025). However, the lethal infection model does not fully recapitulate the clinical observation that increased disease severity but not mortality is the major clinical burden for influenza infected elderly (Kulkarni et al., 2019; Langer et al., 2023). Studies using sublethal infection doses showed that aged mice displayed only modest weight loss following influenza infection (Lu et al., 2018), suggesting that the loss of body weight might not be a reliable symptom indicator under this specific experimental setting. Compared to lethal dose infection, sublethal infection triggers a more controlled and timely antiviral response with limited inflammation, allowing effective viral clearance without causing severe lung pathology in young mice (Brandes et al., 2013; Vogel et al., 2014; Turianová et al., 2019; Ohno et al., 2023).

Based on these considerations, we employed a sublethal influenza infection model to compare pulmonary immune responses between adult (8–12 months) and aged (22–24 months) mice. While body weight loss was comparable, aged mice exhibited impaired viral clearance accompanied by altered innate immune cell recruitment and cell composition. These findings suggest that influenza susceptibility in aged hosts may be driven primarily by defective cellular responses rather than overt clinical manifestations, providing new insights into age-associated impairment of antiviral immunity.

## 2 Results

### 2.1 Body weight changes and viral clearance in mice of different ages after sublethal influenza infection

**Figure 1:**
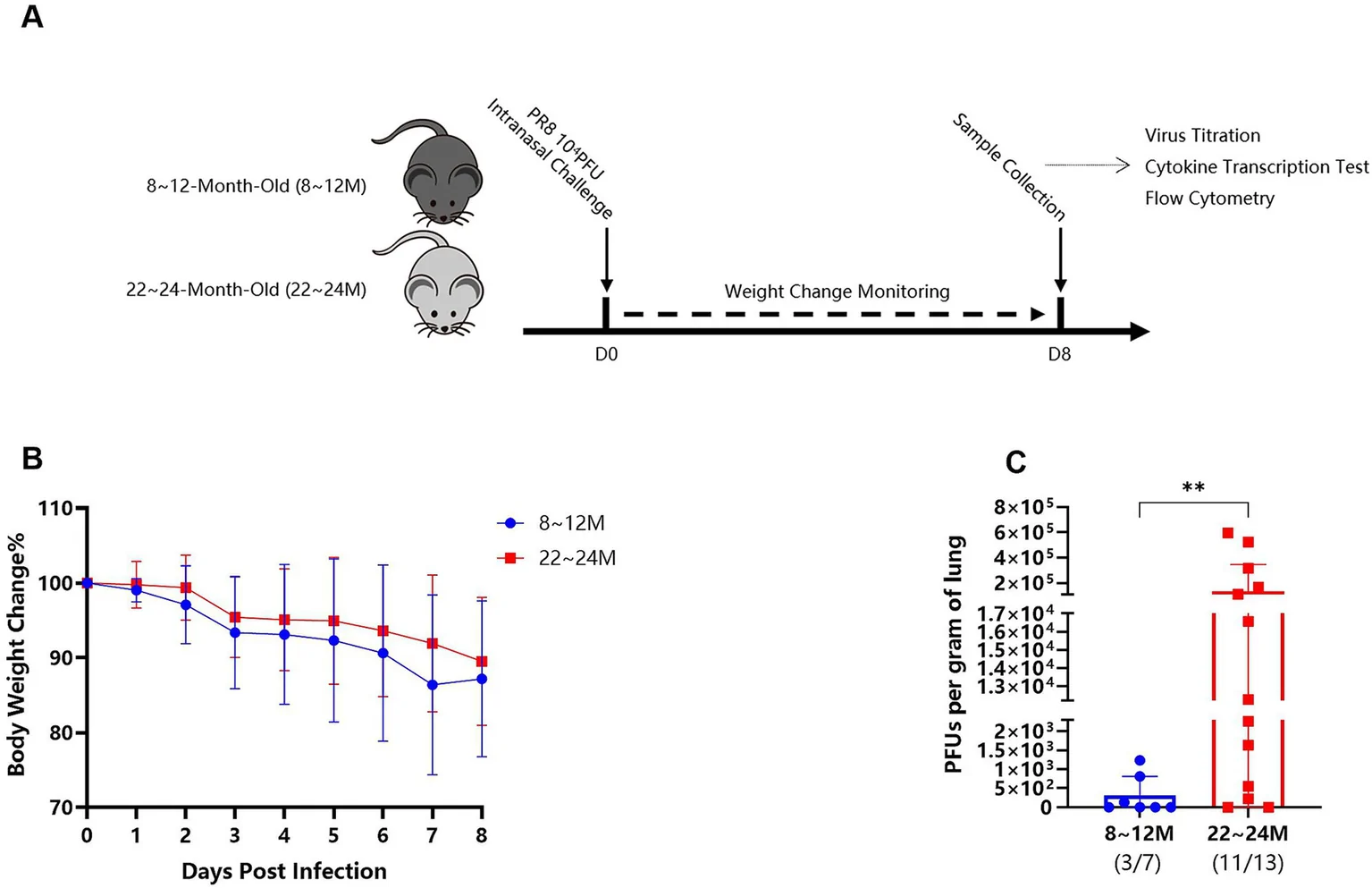
Experimental design and age-dependent differences in body weight and viral clearance following influenza infection. **(A)** The schematic illustration of experimental design. **(B)** Body weight change (%) in 8–12 M and 22–24 M mice following infection, monitored until 8 dpi. Data are shown as mean ± SD. **(C)** Lung viral titers at 8 dpi. Each dot represents an individual mouse, horizontal lines indicate mean ± SD. Numbers below the x-axis indicate virus-persistent mice/total mice in each group. Statistical significance is indicated as follows: *p < 0.05; **p < 0.01; ***p < 0.001.

To delineate the difference of acute responses upon influenza infection between young and aged mice, we firstly explored the establishment of an appropriate infection model through a pilot experiment (Supplementary Figure S1). We found that the manifestation of disease in adolescent (6–8 weeks) mice was much more pronounced than that of young adult (3–5 months) or aged (22–24 months) mice. A previous study suggests that aged mice (16–19 months) are more resistant to influenza infection (Lu et al., 2018). Our observation confirmed this finding and suggested that adult mice are more appropriate to be used as controls while investigating the impact of aging on influenza infection, because they displayed similar clinical susceptibility to influenza infection with aged mice. Based on the above observation, we sought to observe whether and how aging might deviate the acute responses against influenza infection via dissecting viral replication and innate lymphocyte composition in the lungs of adult (8–12 months) and aged mice (22–24 months) at 8 days post infection (Figure 1A).

Our results showed that the average body weights of both groups of mice decreased gradually after infection and were significantly lower on day 8 than day 0 (Figure 1B). While no statistical difference was observed between the dynamic body weight changes of the adult and aged mice, persistent viral replication was detected in a higher proportion of the aged mice (Supplementary Table S1) and the viral titers were significantly higher than those of the adult mice (Figure 1C). These data indicate that, despite similar clinical manifestations, aged mice exhibited impaired viral clearance relative to adult mice.

### 2.2 Aging deviates the correlations of pulmonary innate immune cells with disease severity

To investigate the impact of aging on pulmonary innate immune responses, we first established a multiparameter flow cytometry assay to define major innate immune subsets in mouse lung. As shown in Supplementary Figure S2A, major innate immune cell populations were gated based on established markers, including Ly6C^+^monocytes (Mono, CD45^+^CD11b^+^CD64^−^Ly6C^+^), neutrophils (Neu, CD45^+^CD11b^+^CD64^−^Ly6G^+^), eosinophils (Eos, CD45^+^CD11b^+^CD64^−^SiglecF^+^), alveolar macrophages (AM, CD45^+^CD11b^−^CD64^+^SiglecF^+^), interstitial macrophages (IM, CD45^+^CD11b^+^CD64^+^SiglecF^−^), and transitional monocytes (Trans, CD45^+^CD11b^+^CD64^+^SiglecF^+^).

Using this approach, we first examined the composition of innate immune cells in the lungs of uninfected adult and aged mice. No significant differences were observed in the frequencies of these subsets between the two groups (Supplementary Figure S2B).

**Figure 2:**
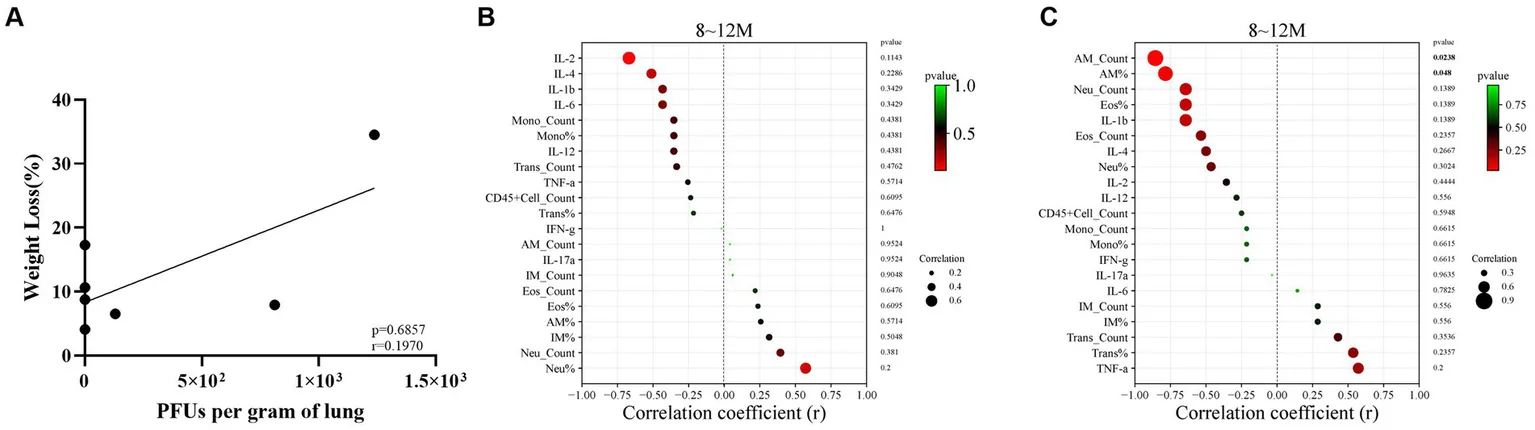
Correlation analysis of viral load, weight loss, innate immune cell subsets, and cytokine responses in adult mice. **(A)** Correlation between lung viral titers and body weight loss at day 8 post infection in adult mice (8–12 months). Each dot represents one mouse. Statistical analysis was performed using Spearman correlation. **(B,C)** Correlation of lung viral titers **(B)** or body weight loss **(C)** with innate immune cell counts, their frequencies (percentage of CD45^+^ cells), and cytokine levels in the lung. Dot size indicates the correlation coefficient (r), and color represents the *p* value (green = higher p, red = lower p). Data are pooled from adult mice at day 8 post infection.

We then applied the same strategy to delineate pulmonary innate immune cell composition at day 8 post infection and analyzed the correlations of these innate immune cell subsets with disease severity. The loss of body weight and the viral titers in the lungs were used as indicators of disease severity. In adult mice, the loss of body weight tended to positively correlate with viral titers, but this association did not reach statistical significance (Figure 2A) partially because the virus had been completely cleared in 4 out of 7 mice. Due to the same reason, we did not observe significant correlations between lung viral titers and the compositions of innate immune cell subsets or the relative transcription levels of inflammatory cytokines (Figure 2B). By contrast, significant negative correlations were found between body weight loss and both the number and frequency of AMs (Figure 2C), which was consistent with previous findings (Schneider et al., 2014) and indicated that AMs play a protective role during influenza infection.

**Figure 3:**
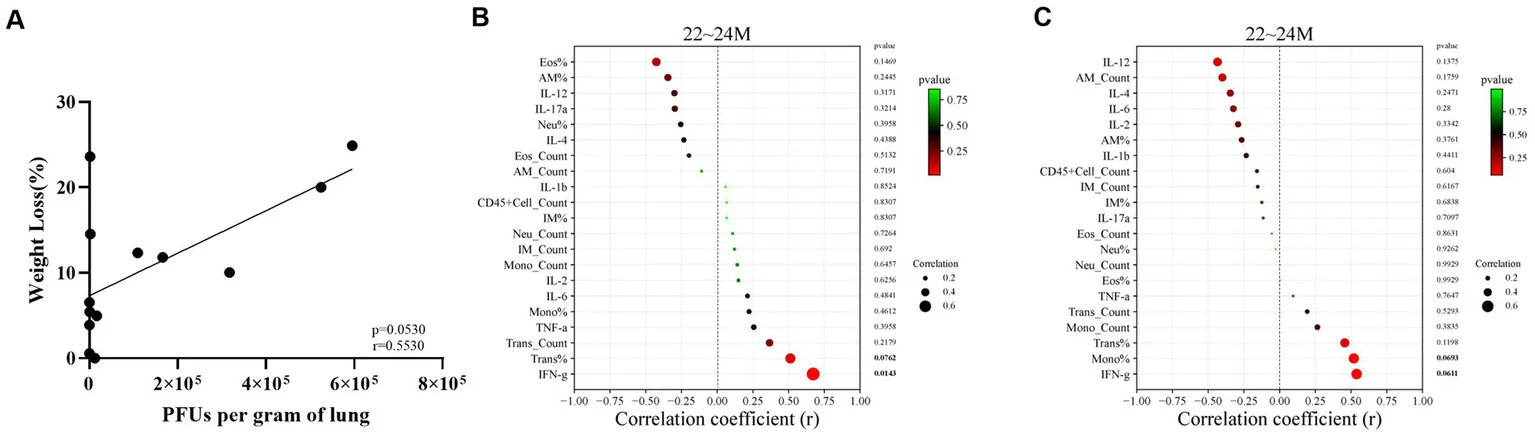
Correlation analysis of viral load, weight loss, innate immune cell subsets, and cytokine responses in aged mice. **(A)** Correlation between lung viral titers and body weight loss at day 8 post infection in aged mice (22–24 months). Each dot represents one mouse. Statistical analysis was performed using Spearman correlation. **(B,C)** Correlation of lung viral titers **(B)** or body weight loss **(C)** with innate immune cell counts, their frequencies (percentage of CD45^+^ cells), and cytokine levels in the lung. Dot size indicates the correlation coefficient (*r*), and color represents the *p* value (green = higher *p*, red = lower *p*). Data are pooled from aged mice at day 8 post infection.

In aged mice, viral titers also tended to positively correlate with body weight loss (*p* = 0.0530) (Figure 3A), while the patterns of correlations between disease severity and the compositions of innate immune cell subsets or the relative transcription levels of inflammatory cytokines were obviously different from those in adult mice. The viral titers in lungs of aged mice significantly correlated with the IFN-*γ* transcription, and also tended to positively correlate with the percentage of transitional monocytes (*p* = 0.0762) (Figure 3B). Meanwhile, despite no significant correlation was observed between the loss of body weight and the measured parameters, a strong tendency toward statistical significance was observed between body weight loss and the frequency of monocytes or IFN-γ transcription level (Figure 3C).

### 2.3 Distinct innate responses between adult and aged mice under persistent viral replication burden

The mice were further stratified into sub-groups according to their viral status at day 8 post infection: adult virus-cleared (Adult-V^−^), adult virus-persistent (Adult-V^+^), aged virus-cleared (Aged-V^−^), and aged virus-persistent (Aged-V^+^). Principal component analyses (PCA) were, respectively, performed using the datasets of innate immune cell composition and transcription levels of cytokines.

**Figure 4:**
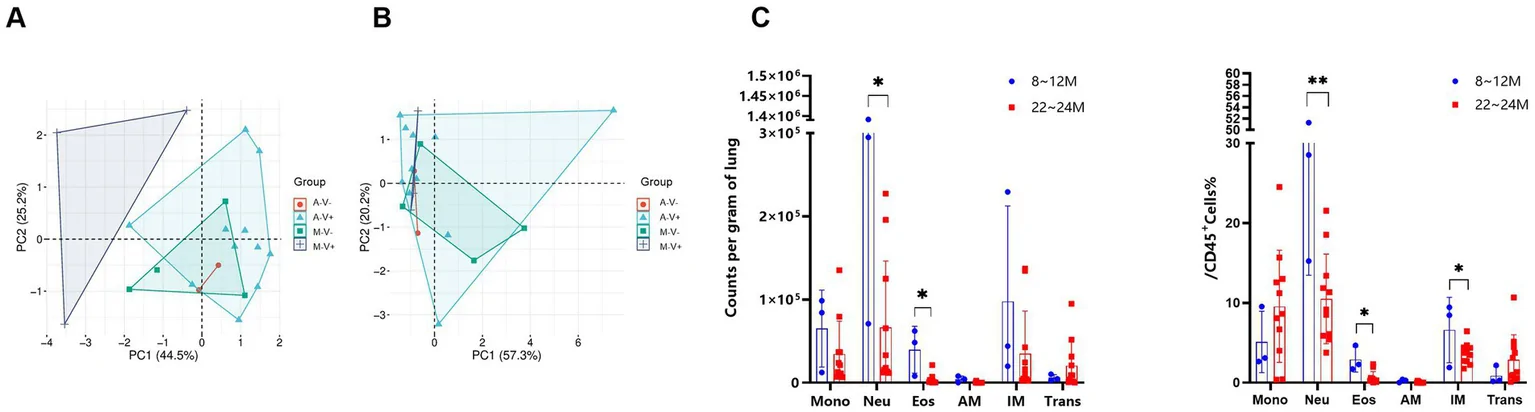
Distinct immune responses between adult and aged mice under persistent viral infection. **(A,B)** Principal component analysis (PCA) of lung immune cell composition **(A)** and cytokine transcriptional profiles **(B)** at day 8 post infection, stratified by age and viral clearance status. Groups include adult-virus-cleared (Adult-V^−^), adult-virus-persistent (Adult-V^+^), aged-virus-cleared (Aged-V^−^), and aged-virus-persistent (Aged-V^+^). **(C)** Comparison of immune cell subsets between Adult-V^+^ and Aged-V^+^ groups. Left: absolute counts per gram of lung tissue; Right: percentage within CD45^+^ cells. Data are presented as mean ± SD. Statistical analysis was performed using unpaired two-tailed Student’s *t* test. Statistical significance is indicated as follows: *p < 0.05; **p < 0.01.

PCA of innate immune cell composition (Figure 4A) revealed that Adult-V^+^ mice were clearly separated from the other three sub-groups, whereas the Aged-V^+^ mice clustered more closely with both the Adult-V^−^ and Aged-V^−^ sub-groups. Quite interestingly, when looking at the PCA of cytokine transcription, we found that all the sub-groups clustered together (Figure 4B). These findings collectively suggested that the innate immune cell might not be efficiently activated in Aged-V^+^ mice although the inflammatory milieu was similar to that of the Adult-V^+^.

Comparisons of innate immune cell composition between Adult-V^+^ and Aged-V^+^ mice further highlighted the following differences: Adult-V^+^ mice displayed significantly higher absolute numbers of neutrophils and eosinophils (Figure 4C left). In terms of proportions, Adult-V^+^ mice exhibited elevated Neu%, IM%, and Eos%, indicating a granulocyte-dominant response (Figure 4C right). In contrast, Aged-V^+^ mice showed relatively enriched macrophage lineages, particularly transitional monocytes. Notably, as shown in Supplementary Figure S3, the total CD45^+^ cell numbers did not differ between adult and aged mice, indicating that these age-associated differences did not arise from impaired lymphocyte infiltration. These findings indicate that age-related differences under viral persistence are primarily reflected in immune cell recruitment and composition rather than cytokine expression, with adult mice mounting a granulocyte-skewed response while aged mice exhibit relative macrophage enrichment.

## 3 Discussion

It is estimated that approximately 20% of the global population will be over 65 years old (Dzau et al., 2019). Many challenges need to be conquered to enable healthy longevity for mankind (Dzau et al., 2020), of which respiratory tract infections represent a serious health problem among elderly individuals (Wang et al., 2024). The senescence of both lung parenchymal cells and immune cells is thought to contribute to the increased mortality and morbidity upon influenza infection in the elderly population (Schneider et al., 2021; Häder et al., 2023; Wang et al., 2024). Using human precision-cut lung slices, a recent study suggests that aged lung tissue does not support optimal replication of influenza and SARS-CoV-2 (Brügger et al., 2025), implying that aged immune system might be a more important factor leading to severe clinical outcomes.

In this study, we tried to delineate the differences of acute responses against infection with a sublethal dose of H1N1 (PR8 strain) between the adult (8–12 months) and aged (22–24 months) mice. We observed a significantly higher average level of viral replication in aged mice at 8 days post infection, but the adult and aged mice displayed similar kinetics of body weight change, which is inconsistent with previous observations (Bartley et al., 2016; Lacasse et al., 2025). This discrepancy may reflect differences in experimental settings. First, we employed a sublethal rather than a lethal infection dose. Lethal models have traditionally been used to investigate mortality in aged hosts, whereas the sublethal infection model may more appropriately mimic the most common situation of real-world infection. Second, unlike earlier investigations that frequently utilized young adult mice (3–4 months) as controls (Toapanta and Ross, 2009; Hernandez-Vargas et al., 2014), we compared middle-aged adult mice (8–12 months) to 22–24-month-old mice. Our study design is based on a pilot experiment demonstrating that 6–8-week-old mice experienced more pronounced weight loss after influenza infection (Supplementary Figure S1). This finding is in accordance with previous studies (Sun et al., 2011; Lu et al., 2018), suggesting that young adult mice might not be proper controls in studying the impact of aging on the consequence of influenza infection. Instead, we propose that middle-aged mice are better controls than young adult mice in this study, because they do not exaggerate the apparent resistance of the aged mice to influenza infection.

Despite the similar body weight loss between the adult and aged mice, we observed that aged mice exhibited significantly elevated pulmonary viral titers at 8 days post infection (Figure 1C). Analogous findings have been reported in clinical settings, where elderly influenza patients frequently display delayed viral clearance (Lee et al., 2009). Moreover, in aged mice, viral titers displayed a near-significant positive correlation with body weight loss (*p* = 0.0530, *r* = 0.5530) (Figure 3A). This finding indicated that viral replication still tended to be manifested by the loss of body weight, but the sensitivity was low. As aged hosts may exhibit immunological hyporesponsiveness and tolerance upon infection or vaccination (Keilich et al., 2019; Goyani et al., 2024), we further measured the pulmonary innate immune cell composition and cytokine transcription to probe whether the acute immune responses were blunted in aged mice. Our data showed that the innate immune cell composition in lung was obviously different between the adult and aged mice. In adult mice, the number and frequency of AMs were significantly and inversely correlated with body weight loss, underscoring the protective role of AMs in mitigating influenza-associated pathology, consistent with prior evidence (Schneider et al., 2014). In aged mice, however, this protective relationship was absent, suggesting that AM function declines with age. Indeed, accumulating evidence indicates that aging profoundly disrupts multiple functional aspects of AM biology. Aged AMs exhibit reduced phagocytic capacity to clear infected or apoptotic epithelial cells (Wong et al., 2017), display broad transcriptional dysregulation that blunts early antiviral signaling (McQuattie-Pimentel et al., 2021; Wang et al., 2024), and show diminished responsiveness to physiological stressors (Boe et al., 2022), leading to delayed or poorly coordinated recruitment of downstream innate immune cells and ultimately to weaker early control of viral replication. Importantly, AM maintenance after influenza infection or other lung injury has been shown to rely not only on self-renewal but also on replenishment by peripheral Ly6C^+^ monocytes, which undergo a transitional differentiation stage to generate monocyte-derived AMs (Trans-AMs) (Li et al., 2022). These transitional cells contribute both to inflammatory responses during recovery and to long-term maintenance of the AM pool and pulmonary homeostasis (Hou et al., 2021). In young mice, disease severity has been attributed primarily to inflammatory responses rather than impaired viral clearance. For example, depletion of transitional monocytes reduces clinical symptoms without affecting pulmonary viral titers (Lin et al., 2011; Coates et al., 2018). In contrast, our study demonstrated that in aged mice, viral titers correlated with the proportion of transitional monocytes, suggesting that the inflammatory and differentiation competence of these monocyte-derived intermediates is diminished with age. This deficiency likely further compromises the restoration and functional capacity of the AM pool, thereby exacerbating viral persistence in aged lungs.

In addition to AMs, we found both the number and the frequency of neutrophils and eosinophils were significantly higher in the virus-persistent adult mice than those in the virus persistent aged mice. Previous studies have identified neutrophils as a central component of the early immune response to influenza, where their appropriate recruitment facilitates viral clearance, but excessive accumulation can exacerbate pulmonary tissue damage (Johansson and Kirsebom, 2021). In mouse models, it has been suggested that exaggerated neutrophil response accounts for the increased mortality in aged mice at the end of influenza infection (Kulkarni et al., 2019). In our study, we did not observe a significant increase of neutrophils at day 8 post infection in aged mice, indicating that neutrophil recruitment under an experimental setting of sublethal influenza infection might be limited even if viral replication is persistent. Aging has also been linked to impaired neutrophil chemotaxis and dysregulated neutrophil trafficking, potentially resulting from dysfunction of the CXCL1–CXCR2 chemotactic axis in aged hosts (Brubaker et al., 2013; Kulkarni et al., 2019; Hornigold et al., 2022). Eosinophils may contribute directly to viral control via releasing RNases, chemokines, and antiviral granule proteins (Sasaki et al., 2025). In animal models, increased eosinophil responses have been associated with reduced weight loss and more rapid viral clearance (Bal et al., 2013; Samarasinghe et al., 2017). Hence, decreased eosinophil recruitment might hamper viral clearance, which could partly explain the persistent viral replication observed in lungs of aged mice. In this study, we did not determine the mechanisms underlying the reduced eosinophil recruitment; however, a human study noted slightly lower mean chemotaxis in older adults without reaching statistical significance (Mathur et al., 2008), suggesting a possible but unconfirmed age-related limitation that could contribute to reduced eosinophil accumulation during infection.

Few limitations should be noted in this study. First, the relatively small number of mice per group may restrict the statistical power for detecting subtle but meaningful differences. Second, we did not validate cytokine responses at the protein level. Third, the primary objective of this study was to characterize age-related differences in viral replication and innate immune profiles under sublethal influenza infection; thus, we did not investigate the underlying mechanisms beyond these phenotypic observations. Nevertheless, our findings highlight the distinct innate immune cell composition in adult and aged mice upon acute influenza infection, which might partially explain impaired viral clearance and delayed recovery in aged hosts. Our findings indicate that even if the clinical symptoms are mild, active virus replication may still occur in aged individuals, underscoring the necessity for extended antiviral treatment.

## 4 Materials and methods

### 4.1 Virus propagation and titration

H1N1 A/Puerto Rico/8/34 (H1N1) (PR8) was propagated in MDCK cells. Briefly, confluent MDCK cells plated in 75 cm^2^ cell culture flasks were inoculated with PR8 at a multiplicity of infection (MOI) of 0.01. After 1-h adsorption at 37 °C with 5% CO_2_, 10 mL of fresh maintenance medium, Dulbecco’s Modified Eagle Medium (DMEM) containing 0.5% bovine serum albumin (BSA), 1% penicillin–streptomycin (PS), 25 mM 4-(2-hydroxyethyl)-1-piperazineethanesulfonic acid (HEPES), and 2 μg/mL TPCK-treated trypsin, was added to each flask. The cell cultures were maintained at 37 °C with 5% CO_2_ for another 3 days. After that, propagated PR8 was harvested from the culture supernatant by centrifuging at 20,000 g for 2 h at 4 °C. Next, the harvested PR8 virus stock was titrated using a plaque assay. Briefly, confluent monolayers of MDCK cells in 12-well plates were infected with 10-fold serial dilutions of the virus stock, ranging from 10^−4^ to 10^−8^. Then, the plates were incubated at 37 °C with 5% CO_2_ for 1 h. After absorption, the inoculum was removed and the cells were covered with 1.5 mL of overlay medium (DMEM containing 0.5% low-melting Agar, 0.5% BSA, 1% PS, 25 mM HEPES and 2 μg/mL TPCK-Trypsin) and incubated at 37 °C with 5% CO2 for 4 days. Post incubation, the overlay medium was removed and the plates were fixed with 2% neutral formalin and stained with 1% crystal violet. Plaques were visually counted, and the viral titers were calculated and expressed as plaque-forming units (PFU) per milliliter.

### 4.2 Mice and *in vivo* viral infection

Experiments using mice were approved by the Research Ethics Review Committee of the Shanghai Public Health Clinical Center Affiliated to Fudan University. Female C57BL/6 J mice were purchased from Shanghai Heyu Biotechnology Co., Ltd. After a 3-day acclimation in the animal biosafety level 2 (ABSL-2) lab, mice were intranasally infected with PR8. Briefly, mice were transiently anesthetized using an animal anesthesia machine (ABS small animal anesthesia machine; Yuyan Instruments, Shanghai, China) with isoflurane (Cat# R510-22, RWD Life Science, China) at 3–4% in oxygen (1.0 L/min) for approximately 1–2 min, until loss of the righting reflex. Subsequently, mice were instilled intranasally with 50 μL phosphate-buffered saline (PBS) containing 1 × 10^4^ PFU of PR8 virus. Following infection, mice were monitored daily for changes of body weight and mortality.

### 4.3 Titration of PR8 in mouse lungs

Eight days post infection, mice were euthanized by cervical dislocation. Lung tissues were then freshly isolated and homogenized using a high-throughput tissue grinding machine (Cat# Scientz-192, NingBo Scientz Biotechnology Co., China). After that, the homogenates were centrifuged at 2,000 g for 10 min and supernatants were collected for viral titration following procedures similar to those described above.

### 4.4 Detection of cytokine transcription in mouse lung

The real-time PCR assay for mouse cytokines was conducted according to the method described in our previous works (Wang et al., 2022; Zhang et al., 2025) with minor modification. Briefly, total RNA was extracted from freshly isolated mouse lung tissues using the Trizol reagent (Cat# 15596018, Invitrogen). 1 μg of total RNA from each sample was reversely transcribed into cDNA using the PrimeScript™ RT Reagent Kit (Cat# RR037A, Takara). The panel of cytokine genes examined by qRT-PCR includes IFN-*γ*, TNF-*α*, IL-2, IL-1β, IL-17a, IL-4, IL-12, and IL-6, with GAPDH serving as the reference gene. The primers of mouse cytokines and GAPDH genes used for qRT-PCR are listed in Supplementary Table S2. Real-time PCR mix was prepared according to the manufacturer’s instructions (TB Green Premix Ex Taq II kit, Cat# RR820A, Takara). PCR reactions were performed using the ABI 7500 Real-Time PCR system (Thermo Fisher Technology Co., USA) under the following conditions: 95 °C pre-denaturation for 30 s, 1 cycle; 95 °C denaturation for 5 s, 60 °C annealing, extension for 34 s, and 40 cycles. Transcription levels of cytokine genes relative to the GAPDH gene were calculated using the 2^−ΔCt^ method, where ΔCt = Ct of the target gene - Ct of GAPDH.

### 4.5 Isolation of the immune cells from mouse lung tissue

Freshly isolated mouse lung tissue was rinsed with sterile PBS and finely minced before being digested with 0.4 mg/mL Collagenase IV (Cat# V900893-1G, Merck) and 0.2 mg/mL DNase I (Cat# 10104159001, Roche) dissolved in Roswell Park Memorial Institute Medium 1,640 (RPMI 1640) at 37 °C in a shaking incubator. 30 min later, digestion was terminated by adding RPMI 1640 containing 5% Fetal Bovine Serum (R5) into the mixture. Next, the digested lung tissue mixture was transferred into 70-μm cell strainers and ground the remaining large aggregates of tissue through the strainer with the rubber end of a syringe plunger. The sieved cell suspension was centrifuged at 400 g for 5 min, and the cell pellet was resuspended in 2 mL red blood cell lysis buffer and incubated for 3 min at room temperature. After incubation, the lysis was stopped by adding 5 mL R5 to each sample and nucleated cells were collected via centrifugation. Finally, purified immune cells were obtained through Percoll density gradient centrifugation. Briefly, 5 mL of 40% isotonic Percoll was added to a 15 mL conical centrifuge tube, and 2 mL of cell suspension was carefully layered on the top. The sample was then centrifuged horizontally at 800 g for 30 min. Cell pellet at the bottom of the tube was collected for downstream flow cytometry assay.

### 4.6 Flow cytometry assay

Firstly, the isolated cells were suspended in 100 μL of PBS buffer and stained with 0.1 μL cell viability dye (Zombie Aqua™ Fixable Viability Kit, Cat# 423101, BioLegend) at room temperature. 15 min later, the staining was stopped by adding RPMI 1640 containing 10% Fetal Bovine Serum (R10) to each sample and the cells were centrifuged at 600 g for 5 min. Cell pellet was resuspended in 50 μL of R10 and stained with fluorochrome-conjugated antibodies for 30 min at 4 °C (CD45 (Brilliant Violet 785™, Cat# 103149, BioLegend); CD11b (Brilliant Violet 605™, Cat# 101257, BioLegend); CD64 (PerCP/Cyanine5.5, Cat# 139307, BioLegend); Ly6G (Alexa Fluor® 488, Cat# 127625, BioLegend); Siglec-F (PE/Cyanine7, Cat# 155527, BioLegend); Ly-6C (Alexa Fluor® 700, Cat# 128024, BioLegend)). After a 30-min incubation, the stained cells were washed with 200 μL R10, and resuspended in R10. Flow cytometry acquisition was performed using a BD LSRFortessa™ Cell Analyzer (BD Biosciences). Data were analyzed with FlowJo™ v10.8.1 software.

### 4.7 Statistical methods

All statistical analyses were performed using GraphPad Prism 9 (GraphPad Software, USA). Data distribution was examined for normality prior to subsequent testing. For two-group comparisons, unpaired t-tests were applied, while differences across multiple groups were assessed using one-way ANOVA. Survival analyses were carried out using the Mantel-Cox log-rank test. A threshold of *p* ≤ 0.05 was considered statistically significant. Principal component analysis (PCA) and correlation plots were generated using https://www.bioinformatics.com.cn (last accessed on 10 December 2024), an online platform for data analysis and visualization.

## Data Availability

The original contributions presented in the study are included in the article/Supplementary material, further inquiries can be directed to the corresponding authors.

## References

[ref1] Allen J. C. Toapanta F. R. Chen W. Tennant S. M. (2020). Understanding immunosenescence and its impact on vaccination of older adults. Vaccine 38, 8264–8272. doi: 10.1016/j.vaccine.2020.11.002, 33229108 PMC7719605

[ref2] Bal S. M. Dijkhuis A. van der Sluijs K. Lutter R. (2013). Antiviral effect of eosinophils in respiratory tract infection with influenza virus in mice. J. Inflamm. 10:P24. doi: 10.1186/1476-9255-10-s1-p24

[ref3] Bartley J. M. Pan S. J. Keilich S. R. Hopkins J. W. Al-Naggar I. M. Kuchel G. A. . (2016). Aging augments the impact of influenza respiratory tract infection on mobility impairments, muscle-localized inflammation, and muscle atrophy. Aging 8, 620–635. doi: 10.18632/aging.100882, 26856410 PMC4925818

[ref4] Boe D. M. Hulsebus H. J. Najarro K. M. Mullen J. E. Kim H. Tan A. C. . (2022). Advanced age is associated with changes in alveolar macrophages and their responses to the stress of traumatic injury. J. Leukoc. Biol. 112, 1371–1386. doi: 10.1002/jlb.3hi0620-399rr, 36120937 PMC10150914

[ref5] Brandes M. Klauschen F. Kuchen S. Germain R. N. (2013). A systems analysis identifies a feedforward inflammatory circuit leading to lethal influenza infection. Cell 154, 197–212. doi: 10.1016/j.cell.2013.06.013, 23827683 PMC3763506

[ref6] Brubaker A. L. Rendon J. L. Ramirez L. Choudhry M. A. Kovacs E. J. (2013). Reduced neutrophil chemotaxis and infiltration contributes to delayed resolution of cutaneous wound infection with advanced age. J. Immunol. 190, 1746–1757. doi: 10.4049/jimmunol.1201213, 23319733 PMC3563860

[ref7] Brügger M. Machahua C. Zumkehr T. Cismaru C. Jandrasits D. Trüeb B. . (2025). Aging shapes infection profiles of influenza a virus and SARS-CoV-2 in human precision-cut lung slices. Respir. Res. 26:112. doi: 10.1186/s12931-025-03190-0, 40128814 PMC11934781

[ref8] Centers for Disease Control and Prevention (2024). Preliminary estimated flu disease burden 2023–2024 flu season. Available online at: https://www.cdc.gov/flu-burden/php/data-vis/2023-2024.html [Accessed 22 July, 2025].

[ref9] Cerqueira-Silva T. Oliveira V. A. Boaventura V. S. Pescarini J. M. Júnior J. B. Machado T. M. . (2022). Influence of age on the effectiveness and duration of protection of Vaxzevria and CoronaVac vaccines: a population-based study. Lancet Reg. Health Am. 6:100154. doi: 10.1016/j.lana.2021.100154, 34957437 PMC8692070

[ref10] Chen J. Deng J. C. Zemans R. L. Bahmed K. Kosmider B. Zhang M. . (2022). Age-induced prostaglandin E(2) impairs mitochondrial fitness and increases mortality to influenza infection. Nat. Commun. 13:6759. doi: 10.1038/s41467-022-34593-y, 36351902 PMC9643978

[ref11] Cipelli R. Falato S. Lusito E. Maifredi G. Montedoro M. Valpondi P. . (2024). The hospital burden of flu in Italy: a retrospective study on administrative data from season 2014-2015 to 2018-2019. BMC Infect. Dis. 24:572. doi: 10.1186/s12879-024-09446-2, 38851739 PMC11162570

[ref12] Coates B. M. Staricha K. L. Koch C. M. Cheng Y. Shumaker D. K. Budinger G. R. S. . (2018). Inflammatory monocytes drive influenza a virus-mediated lung injury in juvenile mice. J. Immunol. 200, 2391–2404. doi: 10.4049/jimmunol.1701543, 29445006 PMC5860989

[ref13] Doherty T. M. Weinberger B. Didierlaurent A. Lambert P. H. (2025). Age-related changes in the immune system and challenges for the development of age-specific vaccines. Ann. Med. 57:2477300. doi: 10.1080/07853890.2025.2477300, 40110678 PMC11926906

[ref14] Dzau V. J. Finkelman E. M. Balatbat C. A. Verdin E. M. Pettigrew R. I. (2020). Achieving healthy human longevity: a global grand challenge. Sci. Transl. Med. 12:3816. doi: 10.1126/scitranslmed.abd3816, 33087500

[ref15] Dzau V. J. Inouye S. K. Rowe J. W. Finkelman E. Yamada T. (2019). Enabling healthful aging for all - the National Academy of medicine grand challenge in healthy longevity. N. Engl. J. Med. 381, 1699–1701. doi: 10.1056/NEJMp1912298, 31633895

[ref16] GBD 2017 Influenza Collaborators (2019). Mortality, morbidity, and hospitalisations due to influenza lower respiratory tract infections, 2017: an analysis for the global burden of disease study 2017. Lancet Respir. Med. 7, 69–89. doi: 10.1016/S2213-2600(18)30496-X, 30553848 PMC6302221

[ref17] Goyani P. Christodoulou R. Vassiliou E. (2024). Immunosenescence: aging and immune system decline. Vaccines 12:1314. doi: 10.3390/vaccines12121314, 39771976 PMC11680340

[ref18] Guo J. Chen X. Guo Y. Liu M. Li P. Tao Y. . (2024). Real-world effectiveness of seasonal influenza vaccination and age as effect modifier: a systematic review, meta-analysis and meta-regression of test-negative design studies. Vaccine 42, 1883–1891. doi: 10.1016/j.vaccine.2024.02.059, 38423813

[ref19] Häder A. Köse-Vogel N. Schulz L. Mlynska L. Hornung F. Hagel S. . (2023). Respiratory infections in the aging lung: implications for diagnosis, therapy, and prevention. Aging Dis. 14, 1091–1104. doi: 10.14336/ad.2023.0329, 37163442 PMC10389836

[ref20] Haq K. McElhaney J. E. (2014). Immunosenescence: influenza vaccination and the elderly. Curr. Opin. Immunol. 29, 38–42. doi: 10.1016/j.coi.2014.03.008, 24769424

[ref21] Hernandez-Vargas E. A. Wilk E. Canini L. Toapanta F. R. Binder S. C. Uvarovskii A. . (2014). Effects of aging on influenza virus infection dynamics. J. Virol. 88, 4123–4131. doi: 10.1128/jvi.03644-13, 24478442 PMC3993746

[ref22] Holtze S. Gorshkova E. Braude S. Cellerino A. Dammann P. Hildebrandt T. B. . (2021). Alternative animal models of aging research. Front. Mol. Biosci. 8:660959. doi: 10.3389/fmolb.2021.660959, 34079817 PMC8166319

[ref23] Hornigold K. Chu J. Y. Chetwynd S. A. Machin P. A. Crossland L. Pantarelli C. . (2022). Age-related decline in the resistance of mice to bacterial infection and in LPS/TLR4 pathway-dependent neutrophil responses. Front. Immunol. 13:888415. doi: 10.3389/fimmu.2022.888415, 36090969 PMC9450589

[ref24] Hou F. Xiao K. Tang L. Xie L. (2021). Diversity of macrophages in lung homeostasis and diseases. Front. Immunol. 12:753940. doi: 10.3389/fimmu.2021.753940, 34630433 PMC8500393

[ref25] Jiang Z. Pan W. Chen Y. Zhou D. Ren S. Tong Q. . (2025). ApoD mediates age-associated increase in vulnerability to influenza virus infection. Proc. Natl. Acad. Sci. USA 122:e2423973122. doi: 10.1073/pnas.2423973122, 40932775 PMC12452933

[ref26] Johansson C. Kirsebom F. C. M. (2021). Neutrophils in respiratory viral infections. Mucosal Immunol. 14, 815–827. doi: 10.1038/s41385-021-00397-4, 33758367 PMC7985581

[ref27] Kasmani M. Y. Topchyan P. Brown A. K. Brown R. J. Wu X. Chen Y. . (2023). A spatial sequencing atlas of age-induced changes in the lung during influenza infection. Nat. Commun. 14:6597. doi: 10.1038/s41467-023-42021-y, 37852965 PMC10584893

[ref28] Keilich S. R. Bartley J. M. Haynes L. (2019). Diminished immune responses with aging predispose older adults to common and uncommon influenza complications. Cell. Immunol. 345:103992. doi: 10.1016/j.cellimm.2019.103992, 31627841 PMC6939636

[ref29] Kulkarni U. Zemans R. L. Smith C. A. Wood S. C. Deng J. C. Goldstein D. R. (2019). Excessive neutrophil levels in the lung underlie the age-associated increase in influenza mortality. Mucosal Immunol. 12, 545–554. doi: 10.1038/s41385-018-0115-3, 30617300 PMC6375784

[ref30] Lacasse É. Dubuc I. Gudimard L. Andrade A. Gravel A. Greffard K. . (2025). Delayed viral clearance and altered inflammatory responses affect severity of SARS-CoV-2 infection in aged mice. Immun. Ageing 22:11. doi: 10.1186/s12979-025-00503-1, 40075368 PMC11899864

[ref31] Langer J. Welch V. L. Moran M. M. Cane A. Lopez S. M. C. Srivastava A. . (2023). High clinical burden of influenza disease in adults aged ≥ 65 years: can we do better? A systematic literature review. Adv. Ther. 40, 1601–1627. doi: 10.1007/s12325-023-02432-1, 36790682 PMC9930064

[ref32] Lee N. Chan P. K. Hui D. S. Rainer T. H. Wong E. Choi K. W. . (2009). Viral loads and duration of viral shedding in adult patients hospitalized with influenza. J. Infect. Dis. 200, 492–500. doi: 10.1086/600383, 19591575 PMC7110250

[ref33] Lee K. A. Flores R. R. Jang I. H. Saathoff A. Robbins P. D. (2022). Immune senescence, immunosenescence and aging. Front. Aging 3:900028. doi: 10.3389/fragi.2022.900028, 35821850 PMC9261375

[ref34] Li F. Piattini F. Pohlmeier L. Feng Q. Rehrauer H. Kopf M. (2022). Monocyte-derived alveolar macrophages autonomously determine severe outcome of respiratory viral infection. Sci. Immunol. 7:eabj5761. doi: 10.1126/sciimmunol.abj5761, 35776802

[ref35] Lin K. L. Sweeney S. Kang B. D. Ramsburg E. Gunn M. D. (2011). CCR2-antagonist prophylaxis reduces pulmonary immune pathology and markedly improves survival during influenza infection. J. Immunol. 186, 508–515. doi: 10.4049/jimmunol.1001002, 21098218 PMC3723340

[ref36] Liu Z. Liang Q. Ren Y. Guo C. Ge X. Wang L. . (2023). Immunosenescence: molecular mechanisms and diseases. Signal Transduct. Target. Ther. 8:200. doi: 10.1038/s41392-023-01451-2, 37179335 PMC10182360

[ref37] Lu J. Duan X. Zhao W. Wang J. Wang H. Zhou K. . (2018). Aged mice are more resistant to influenza virus infection due to reduced inflammation and lung pathology. Aging Dis. 9, 358–373. doi: 10.14336/ad.2017.0701, 29896425 PMC5988592

[ref38] Mathur S. K. Schwantes E. A. Jarjour N. N. Busse W. W. (2008). Age-related changes in eosinophil function in human subjects. Chest 133, 412–419. doi: 10.1378/chest.07-2114, 18252914 PMC2919352

[ref39] McQuattie-Pimentel A. C. Ren Z. Joshi N. Watanabe S. Stoeger T. Chi M. . (2021). The lung microenvironment shapes a dysfunctional response of alveolar macrophages in aging. J. Clin. Invest. 131:299. doi: 10.1172/jci140299, 33586677 PMC7919859

[ref40] Murasko D. M. Bernstein E. D. Gardner E. M. Gross P. Munk G. Dran S. . (2002). Role of humoral and cell-mediated immunity in protection from influenza disease after immunization of healthy elderly. Exp. Gerontol. 37, 427–439. doi: 10.1016/s0531-5565(01)00210-8, 11772530

[ref41] Nguyen A. M. Noymer A. (2013). Influenza mortality in the United States, 2009 pandemic: burden, timing and age distribution. PLoS One 8:e64198. doi: 10.1371/journal.pone.0064198, 23717567 PMC3661470

[ref42] Ohno M. Gowda S. G. B. Sekiya T. Nomura N. Shingai M. Hui S. P. . (2023). The elucidation of plasma lipidome profiles during severe influenza in a mouse model. Sci. Rep. 13:14210. doi: 10.1038/s41598-023-41055-y, 37648726 PMC10469212

[ref43] Paget J. Spreeuwenberg P. Charu V. Taylor R. J. Iuliano A. D. Bresee J. . (2019). Global mortality associated with seasonal influenza epidemics: new burden estimates and predictors from the GLaMOR project. J. Glob. Health 9:020421. doi: 10.7189/jogh.09.020421, 31673337 PMC6815659

[ref44] Riese P. Trittel S. Akmatov M. K. May M. Prokein J. Illig T. . (2022). Distinct immunological and molecular signatures underpinning influenza vaccine responsiveness in the elderly. Nat. Commun. 13:6894. doi: 10.1038/s41467-022-34487-z, 36371426 PMC9653450

[ref45] Samarasinghe A. E. Melo R. C. Duan S. LeMessurier K. S. Liedmann S. Surman S. L. . (2017). Eosinophils promote antiviral immunity in mice infected with influenza a virus. J. Immunol. 198, 3214–3226. doi: 10.4049/jimmunol.1600787, 28283567 PMC5384374

[ref46] Sasaki H. Miyata J. Kawana A. Fukunaga K. (2025). Antiviral roles of eosinophils in asthma and respiratory viral infection. Front. Allergy 6:1548338. doi: 10.3389/falgy.2025.1548338, 40083723 PMC11903450

[ref47] Schneider C. Nobs S. P. Heer A. K. Kurrer M. Klinke G. van Rooijen N. . (2014). Alveolar macrophages are essential for protection from respiratory failure and associated morbidity following influenza virus infection. PLoS Pathog. 10:e1004053. doi: 10.1371/journal.ppat.1004053, 24699679 PMC3974877

[ref48] Schneider J. L. Rowe J. H. Garcia-de-Alba C. Kim C. F. Sharpe A. H. Haigis M. C. (2021). The aging lung: physiology, disease, and immunity. Cell 184, 1990–2019. doi: 10.1016/j.cell.2021.03.005, 33811810 PMC8052295

[ref49] Sun S. Zhao G. Xiao W. Hu J. Guo Y. Yu H. . (2011). Age-related sensitivity and pathological differences in infections by 2009 pandemic influenza a (H1N1) virus. Virol. J. 8:52. doi: 10.1186/1743-422x-8-52, 21299904 PMC3041774

[ref50] Toapanta F. R. Ross T. M. (2009). Impaired immune responses in the lungs of aged mice following influenza infection. Respir. Res. 10:112. doi: 10.1186/1465-9921-10-112, 19922665 PMC2785782

[ref51] Turianová L. Lachová V. Svetlíkova D. Kostrábová A. Betáková T. (2019). Comparison of cytokine profiles induced by nonlethal and lethal doses of influenza a virus in mice. Exp. Ther. Med. 18, 4397–4405. doi: 10.3892/etm.2019.8096, 31777543 PMC6862669

[ref52] Vogel A. J. Harris S. Marsteller N. Condon S. A. Brown D. M. (2014). Early cytokine dysregulation and viral replication are associated with mortality during lethal influenza infection. Viral Immunol. 27, 214–224. doi: 10.1089/vim.2013.0095, 24787235 PMC4043423

[ref53] Wang Y. Huang X. Luo G. Xu Y. Deng X. Lin Y. . (2024). The aging lung: microenvironment, mechanisms, and diseases. Front. Immunol. 15:1383503. doi: 10.3389/fimmu.2024.1383503, 38756780 PMC11096524

[ref54] Wang X. Jia L. Liu Y. Wang J. Qiu C. Li T. . (2022). Immune correlates of disseminated BCG infection in IL12RB1-deficient mice. Vaccines 10:1147. doi: 10.3390/vaccines10071147, 35891311 PMC9316795

[ref55] Wong C. K. Smith C. A. Sakamoto K. Kaminski N. Koff J. L. Goldstein D. R. (2017). Aging impairs alveolar macrophage phagocytosis and increases influenza-induced mortality in mice. J. Immunol. 199, 1060–1068. doi: 10.4049/jimmunol.1700397, 28646038 PMC5557035

[ref56] Yang J. Ren W. Ren Y. Yu T. Ali L. (2025). Potential of biomarkers of ageing in predicting severity of influenza virus infection and vaccination efficacy. NPJ Aging 11:56. doi: 10.1038/s41514-025-00212-5, 40595702 PMC12217183

[ref57] Zhang Y. Wan Y. Guo C. Zhu Z. Qiu C. Lu J. . (2025). Novel derivatives of brincidofovir and (S)-9-(3-hydroxy-2-phosphonylmethoxypropyl)adenine inhibit orthopoxviruses and human adenoviruses more potently than brincidofovir. Signal Transduct. Target. Ther. 10:114. doi: 10.1038/s41392-025-02207-w, 40210872 PMC11985979

