# Correction: Skewed pulmonary innate immune cell composition underlies the delayed influenza clearance in aged mice

**DOI:** 10.3389/fmicb.2026.1973221

**Published:** 2026-09-08

**Authors:** Xiaoyang Cheng, Fang Zhao, Jing Wu, Yanmin Wan, Zhaoqin Zhu, Jialin Jin

**Affiliations:** 1 Department of Infectious Diseases, Shanghai Key Laboratory of Infectious Diseases and Biosafety Emergency Response, National Medical Center for Infectious Diseases, Huashan Hospital, Shanghai Medical College, Fudan University, Shanghai, China; 2 Department of Laboratory Medicine, Shanghai Public Health Clinical Center, Shanghai, China; 3 Department of Radiology, Shanghai Xuhui Central Hospital, Shanghai, China; 4 Department of Radiology, Shanghai Public Health Clinical Center, Shanghai, China; 5 Shanghai Sci-Tech Inno Center for Infection and Immunity, Shanghai, China

**Keywords:** aging, influenza, sublethal infection, innate immune cell, inflammatory cytokine

There was a mistake in Figure 4, page 5 as published.

The group labels in panels A and B were incorrect. Specifically, “A-V–,” “A-V+,” “M-V–,” and “M-V+” should read “Aged-V–,” “Aged-V+,” “Adult-V–,” and “Adult-V+,” respectively. The figure legend was correct and remains unchanged.

The corrected Figure 4 appears below.

**Figure 4:**
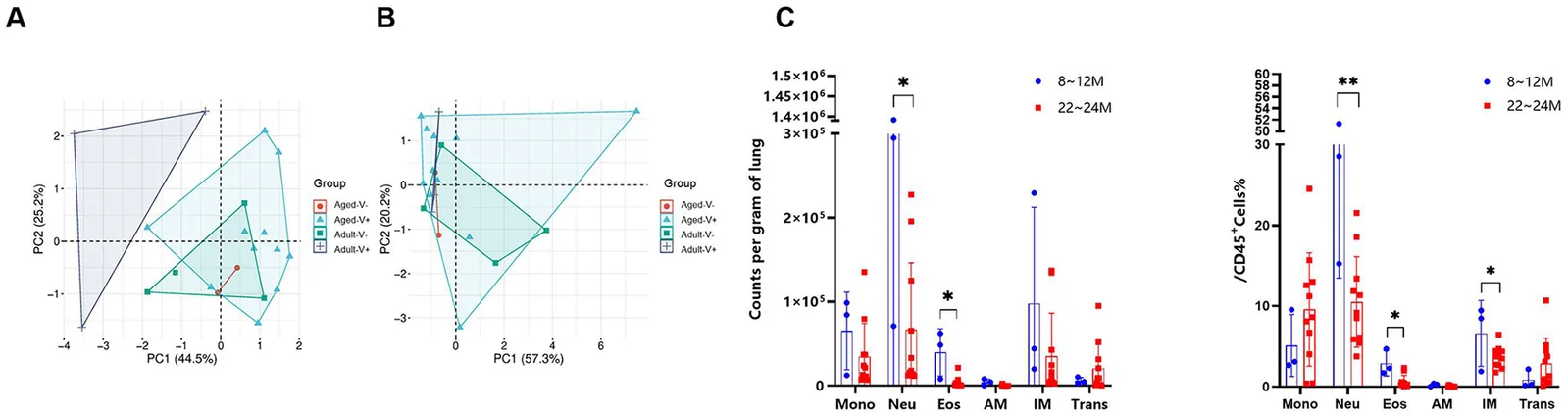
Distinct immune responses between adult and aged mice under persistent viral infection. (**A, B**) Principal component analysis (PCA) of lung immune cell composition (**A**) and cytokine transcriptional profiles (**B**) at day 8 post infection, stratified by age and viral clearance status. Groups include adult-virus-cleared (Adult-V−), adult-virus-persistent (Adult-V^+^), aged-virus-cleared (Aged-V^−^), and aged-virus-persistent (Aged-V^+^). (**C**) Comparison of immune cell subsets between Adult-V^+^ and Aged-V^+^ groups. Left: absolute counts per gram of lung tissue; Right: percentage within CD45^+^ cells. Data are presented as mean ± SD. Statistical analysis was performed using unpaired two-tailed Student’s t test. Statistical significance is indicated as follows: ^*^p < 0.05; ^**^p < 0.01.

The original version of this article has been updated.

