# Supplementary material for: Skewed pulmonary innate immune cell composition underlies the delayed influenza clearance in aged mice
Source: Front Microbiol. 2025 Dec 10;16:1734163. doi: 10.3389/fmicb.2025.1734163 (PMC12727561; doi:10.3389/fmicb.2025.1734163)
Supplement: Supplementary file 1 [file Data_Sheet_1.docx]

Supplementary Material

# Supplementary Figures


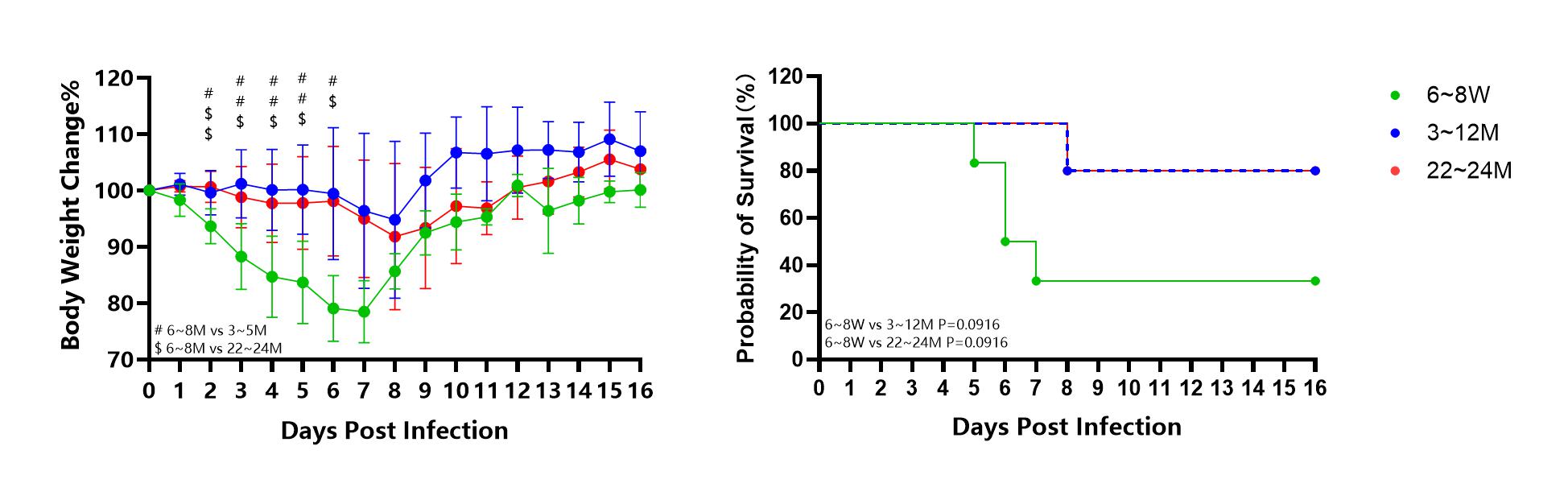


**Figure S1.** Body weight dynamics and survival in mice of different ages. Weight loss and survival rates of mice after being intranasally infected with 10,000 PFU of PR8 virus. Weight loss ≥25% of initial body weight was recorded as ethical death and was therefore not represented in the figure. The data of weight loss are presented as mean ± SD. Statistical significance is indicated as follows: $, #: p < 0.05; $$, ##: p < 0.01.


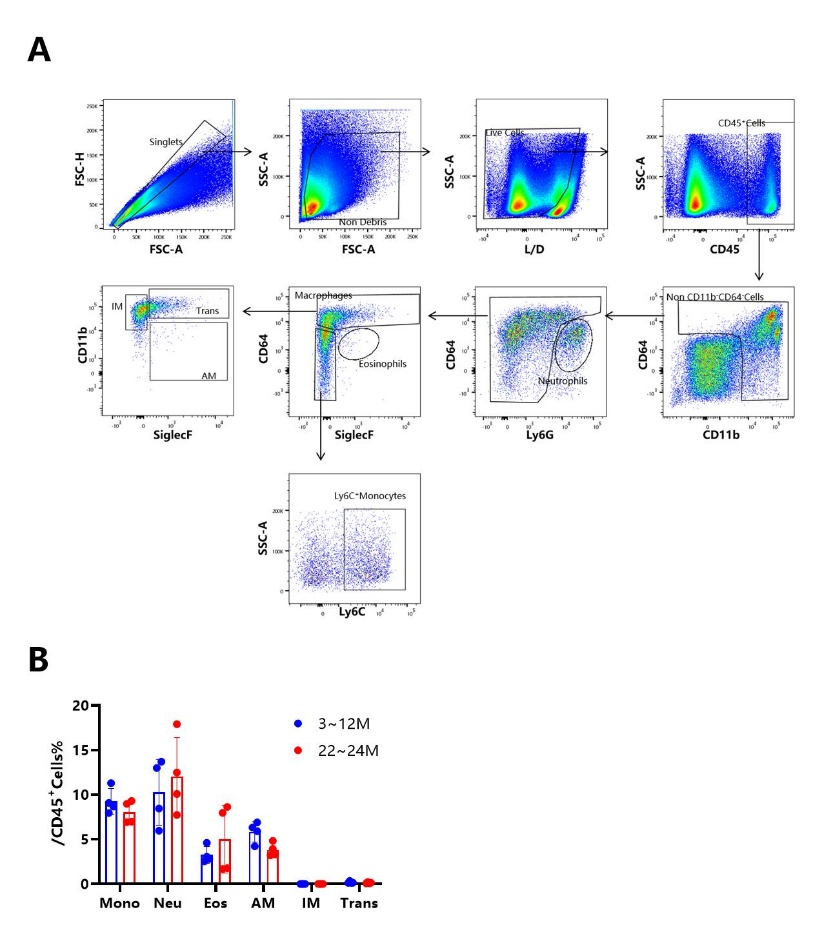


**Figure S2.** Gating strategy and baseline distribution of pulmonary innate immune cell subsets. (A) Representative flow cytometry gating strategy for the identification of major innate immune cell subsets in mouse lungs. Single cells were gated based on FSC-A vs. FSC-H, followed by ex-clusion of debris and dead cells. Live CD45⁺ leukocytes were subsequently analyzed to define the following populations: alveolar macrophages (AM, CD45⁺CD11b⁻CD64⁺SiglecF⁺), interstitial macrophages (IM, CD45⁺CD11b⁺CD64⁺SiglecF⁻), transitional monocytes (Trans, CD45⁺CD11b⁺CD64⁺SiglecF⁺), neutrophils (Neu, CD45⁺CD11b⁺CD64⁻Ly6G⁺), eosinophils (Eos, CD45⁺CD11b⁺CD64⁻SiglecF⁺), and Ly6C⁺ monocytes (Mono, CD45⁺CD11b⁺CD64⁻Ly6C⁺). (B) Base-line frequencies of innate immune cell subsets among total CD45⁺ cells in the lungs of adult (8–12 months) and aged (22–24 months) mice. Data are shown as mean ± SD, with each dot representing one individual mouse.


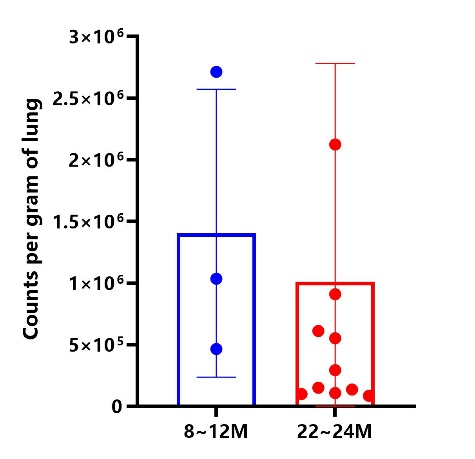


**Figure S3.** Absolute numbers of CD45⁺ lung immune cells in virus-persistent adult and aged mice. Absolute CD45⁺ cell counts per gram of lung tissue in adult (8–12 months) and aged (22–24 months) mice with persistent viral replication at day 8 post-infection. Each dot represents one mouse; bars show mean ± SD.

# Supplementary Tables

**Supplementary Table 1.** Comparison of viral clearance between adult (8–12 M) and aged (22–24 M) mice at day 8 post in-fluenza infection

| **Age group** | **Virus-persistent (V+)** | **Virus-cleared (V-)** | **Total** |
| --- | --- | --- | --- |
| 8-12M | 3 | 4 | 7 |
| 22-24M | 11 | 2 | 13 |
| Total | 14 | 6 | 20 |

Statistical analysis: Fisher’s exact test (two-tailed): p = 0.1219 (ns); Relative Risk (RR) = 0.51 (95% CI: 0.18–1.01); Odds Ratio (OR) = 0.14 (95% CI: 0.02–1.12).

**Supplementary Table 2.** Primer sequences used for quantitative PCR assays of murine cytokines

| **Gene** |  | **Primer sequence 5' to 3'** |
| --- | --- | --- |
| GAPDH | FP | ACCCAGAAGACTGTGGATGG |
|  | RP | GGATGCAGGGATGATGTTCT |
| IFN-g | FP | ACAGCAAGGCGAAAAAGGATG |
|  | RP | TGGTGGACCACTCGGATGA |
| TNF-a | FP | CATCTTCTCAAAATTCGAGTGACAA |
|  | RP | TGGGAGTAGACAAGGTACAACCC |
| IL-2 | FP | TCTGCGGCATGTTCTGGATTT |
|  | RP | ATGTGTTGTCAGAGCCCTTTAG |
| IL-1b | FP | CAACCAACAAGTGATATTCTCCATG |
|  | RP | GATCCACACTCTCCAGCTGCA |
| IL-17a | FP | GGCCCTCAGACTACCTCAAC |
|  | RP | TCTCGACCCTGAAAGTGAAGG |
| IL-4 | FP | ATCGGCATTTTGAACGAGGTC |
|  | RP | GAGGACGTTTGGCACATCCA |
| IL-12 | FP | CTGTGCCTTGGTAGCATCTATG |
|  | RP | CGCAGAGTCTCGCCATTATGAT |
| IL-6 | FP | GAGGATACCACTCCCAACAGACC |
|  | RP | AAGTGCATCATCGTTGTTCATACA |

FP: Forward primer; RP: Reverse primer
